# Supplementary material for: Deep transcriptomics reveals cell-specific isoforms of pan-neuronal genes
Source: Nat Commun. 2025 May 16;16:4507. doi: 10.1038/s41467-025-58296-2 (PMC12084633; doi:10.1038/s41467-025-58296-2)
Supplement: Supplementary file 1 — Supplementary Information [file 41467_2025_58296_MOESM1_ESM.pdf]

SUPPLEMENTARY FIGURES

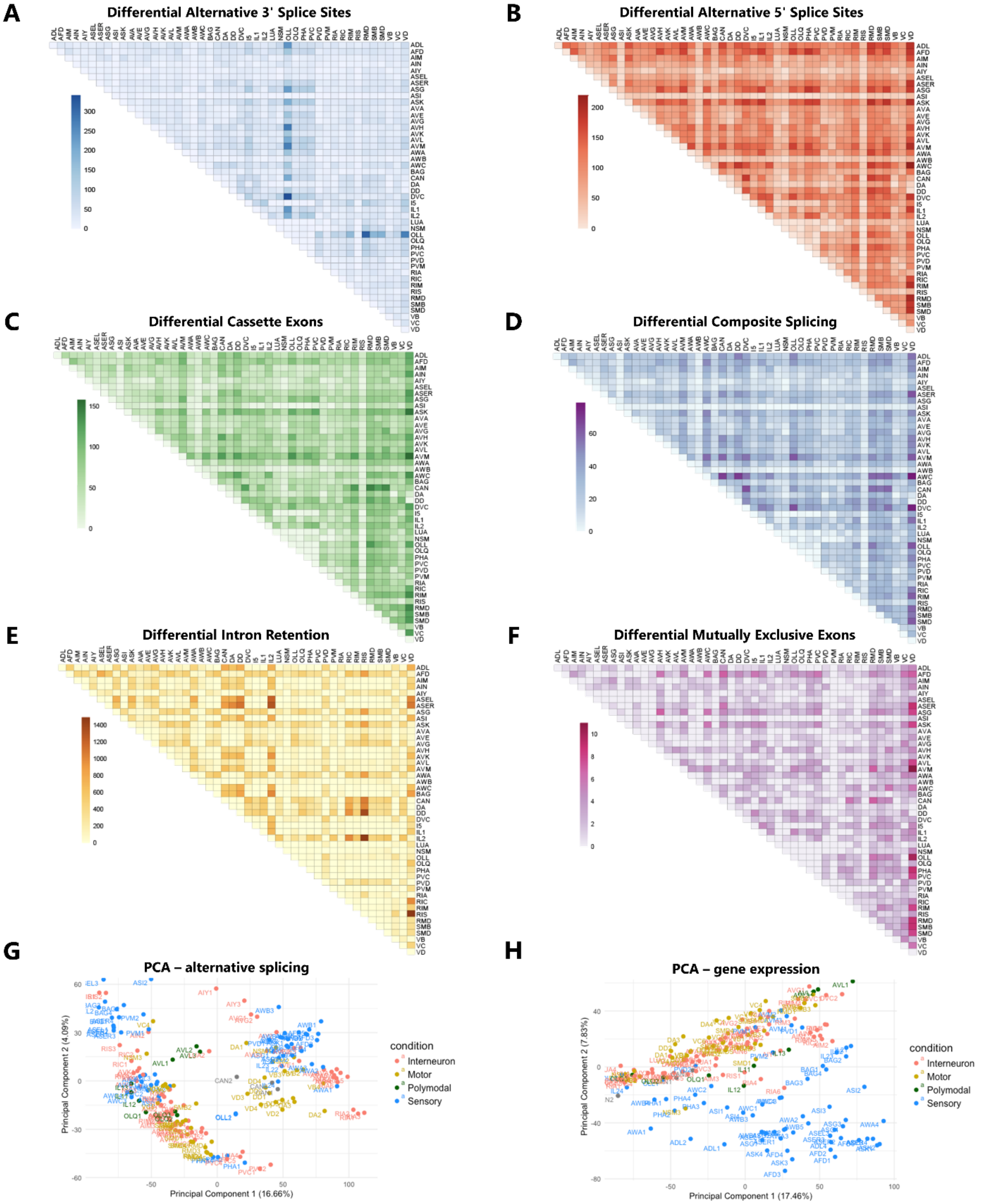

Supplementary Fig. 1: Alternative splicing across neuronal cell types revealed by deep transcriptomes. (A-F) Heatmaps, as in Figure 1E, showing number of differential alternative splicing events across all pairwise comparisons for all classes of alternative splicing detectable by JUM: (A) alternative 3′ splice site, (B) alternative 5′ splice site, (C) cassette exons, (D) composite splicing, (E) intron retention, and (F) mutually exclusive exons. (G & H) Principal component analyses using data from either (G) cell-specific splicing values (excluding composite values due to their complex nature) or (H) gene expression values show alternative clustering patterns, suggesting orthogonal modes of regulation.

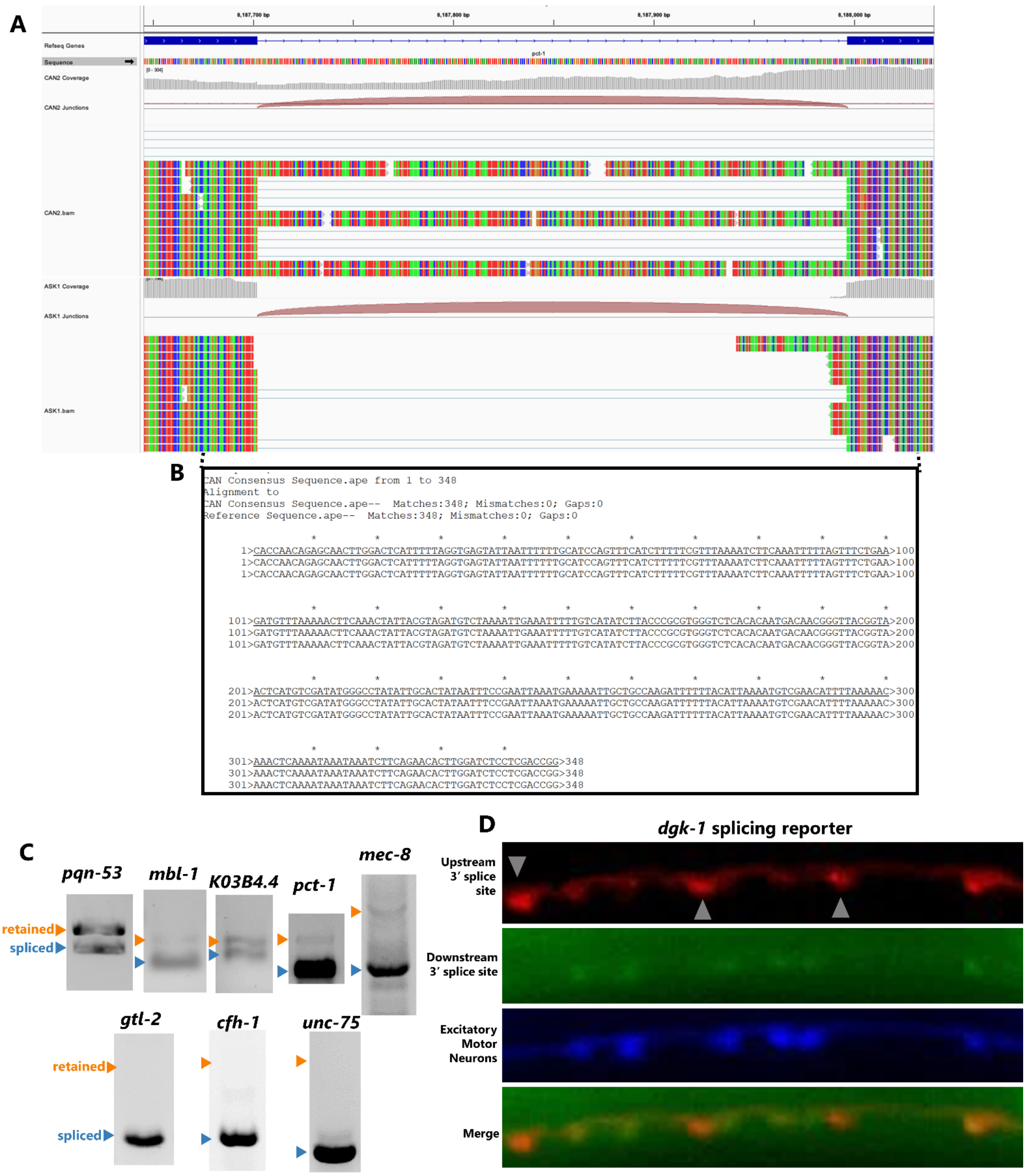
A
8,187,700 bp
8,187,800 bp
8,187,900 bp
8,188,000 bp
Refseq Genes
Sequence
pct-1
CAN2 Coverage
CAN2 Junctions
CAN2.bam
ASK1 Coverage
ASK1 Junctions
ASK1.bam
B
CAN Consensus Sequence.ape from 1 to 348
Alignment to
CAN Consensus Sequence.ape-- Matches:348; Mismatches:0; Gaps:0
Reference Sequence.ape-- Matches:348; Mismatches:0; Gaps:0
1>CACCAACAGAGCAACTTGGACTCATTTTTAGGTGAGTATTAATTTTTTGCATCCAGTTTCATCTTTTTCGTTTAAAATCTTCAAATTTTTAGTTTCTGAA>100
1>CACCAACAGAGCAACTTGGACTCATTTTTAGGTGAGTATTAATTTTTTGCATCCAGTTTCATCTTTTTCGTTTAAAATCTTCAAATTTTTAGTTTCTGAA>100
1>CACCAACAGAGCAACTTGGACTCATTTTTAGGTGAGTATTAATTTTTTGCATCCAGTTTCATCTTTTTCGTTTAAAATCTTCAAATTTTTAGTTTCTGAA>100
101>GATGTTTAAAAACTTCAAACTATTACGTAGATGTCTAAAATTGAAATTTTTGTCATATCTTACCCGCGTGGGTCTCACACAATGACAACGGGTTACGGTA>200
101>GATGTTTAAAAACTTCAAACTATTACGTAGATGTCTAAAATTGAAATTTTTGTCATATCTTACCCGCGTGGGTCTCACACAATGACAACGGGTTACGGTA>200
101>GATGTTTAAAAACTTCAAACTATTACGTAGATGTCTAAAATTGAAATTTTTGTCATATCTTACCCGCGTGGGTCTCACACAATGACAACGGGTTACGGTA>200
201>ACTCATGTCGATATGGGCCTATATTGCACTATAATTTCCGAATTAAATGAAAAATTGCTGCCAAGATTTTTTACATTAAAATGTCGAACATTTTAAAAAC>300
201>ACTCATGTCGATATGGGCCTATATTGCACTATAATTTCCGAATTAAATGAAAAATTGCTGCCAAGATTTTTTACATTAAAATGTCGAACATTTTAAAAAC>300
201>ACTCATGTCGATATGGGCCTATATTGCACTATAATTTCCGAATTAAATGAAAAATTGCTGCCAAGATTTTTTACATTAAAATGTCGAACATTTTAAAAAC>300
301>AAACTCAAAATAAATAAATCTTCAGAACACTTGGATCTCCTCGACCGG>348
301>AAACTCAAAATAAATAAATCTTCAGAACACTTGGATCTCCTCGACCGG>348
301>AAACTCAAAATAAATAAATCTTCAGAACACTTGGATCTCCTCGACCGG>348
C
pqn-53
mbl-1
K03B4.4
pct-1
mec-8
retained
spliced
gtl-2
cfh-1
unc-75
retained
spliced
D
dgk-1 splicing reporter
Upstream 3' splice site
Downstream 3' splice site
Excitatory Motor Neurons
Merge

E

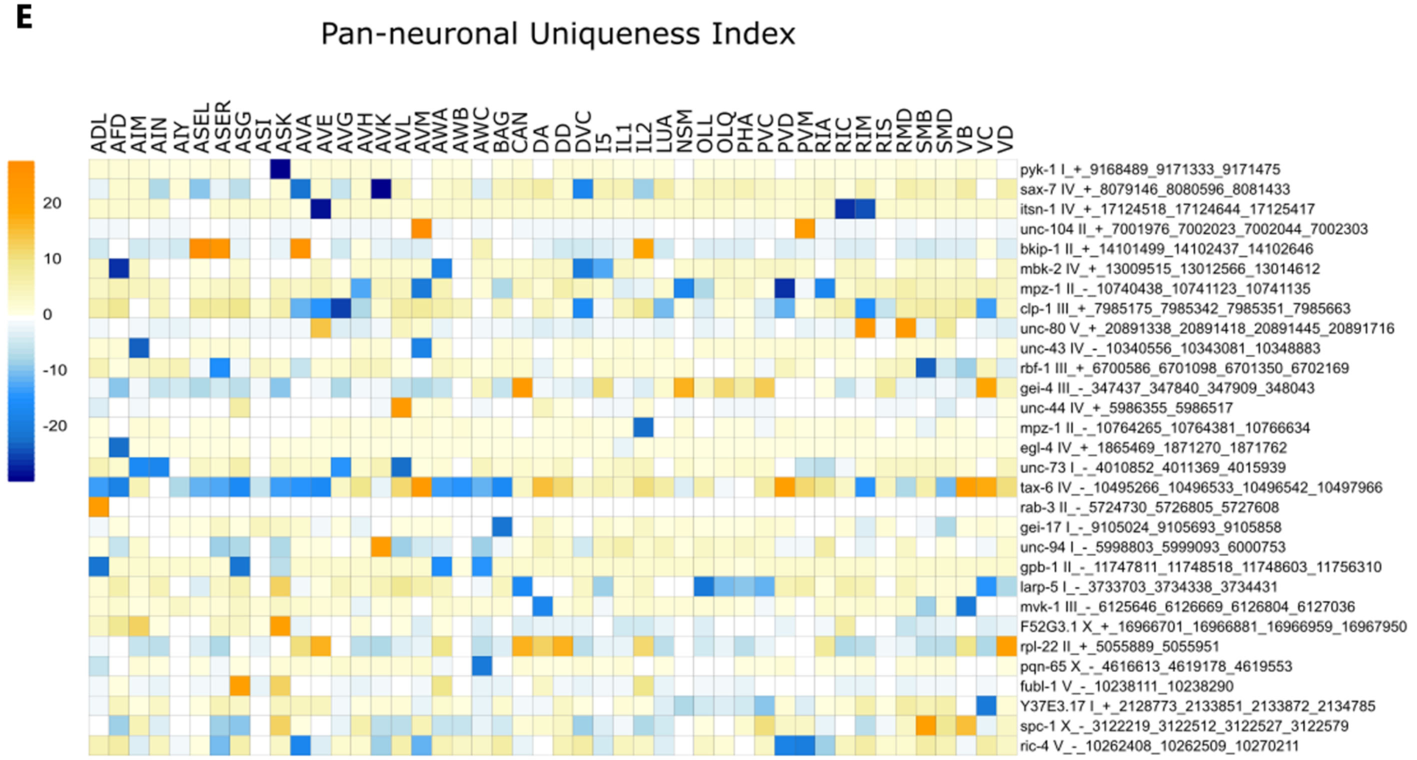

F

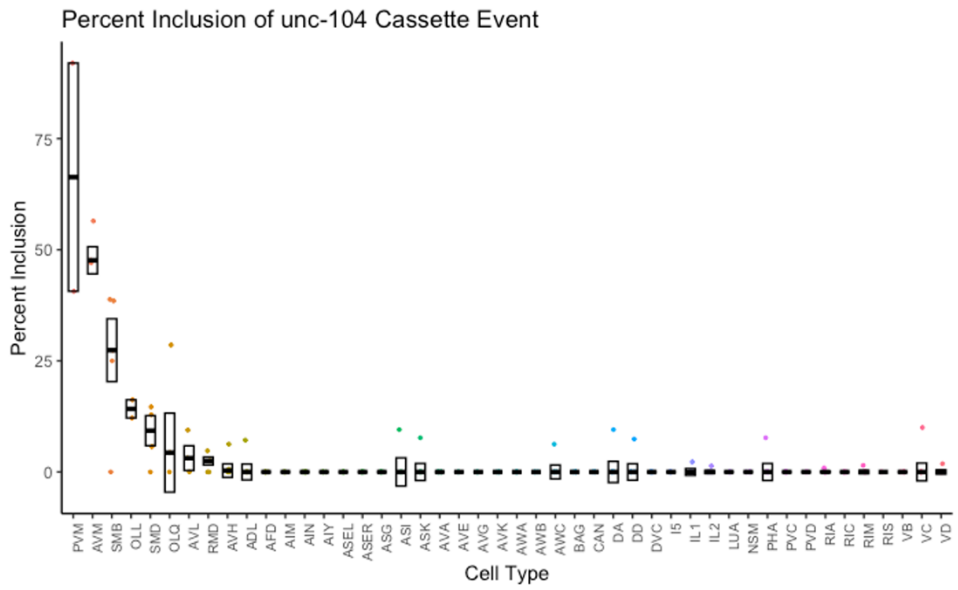

Supplementary Fig. 2: Validation of alternative intron retention events and pan-neuronal genes with cell-specific isoforms. (A) Visualization of sequencing reads via Integrative Genomics Viewer (IGV) showing no mutations in either CAN or ASK neuron RNA Seq (lower tracks) compared to each other, or to reference sequence (upper track). (B) Sequence alignment between the annotated nucleotides and intron-retained sequenced nucleotides showing no mutations observed. (C) RT-PCR validation of five retained introns (selected among the top 20% of uniqueness index). All (5/5) introns tested revealed evidence for both spliced and retained intron products. Below are 3 control introns not predicted to be retained in appreciable amounts. (D) Zoomed in micrograph of Figure 2D; gray arrows denote GABAergic neurons, which do not express the

downstream (GFP) isoform. (E) Highest uniqueness values for pan-neuronal genes defined as displaying single-cell gene expression in at least 123/126 cell types from the CeNGEN single-neuron data. This cutoff was selected as the median number of neurons in which experimentally-defined pan-neuronal genes are expressed (Stefanakis *et al.* 2015). (F) Raw PSI values for pan-neuronal gene *unc-104* across all replicates of all neuron types.

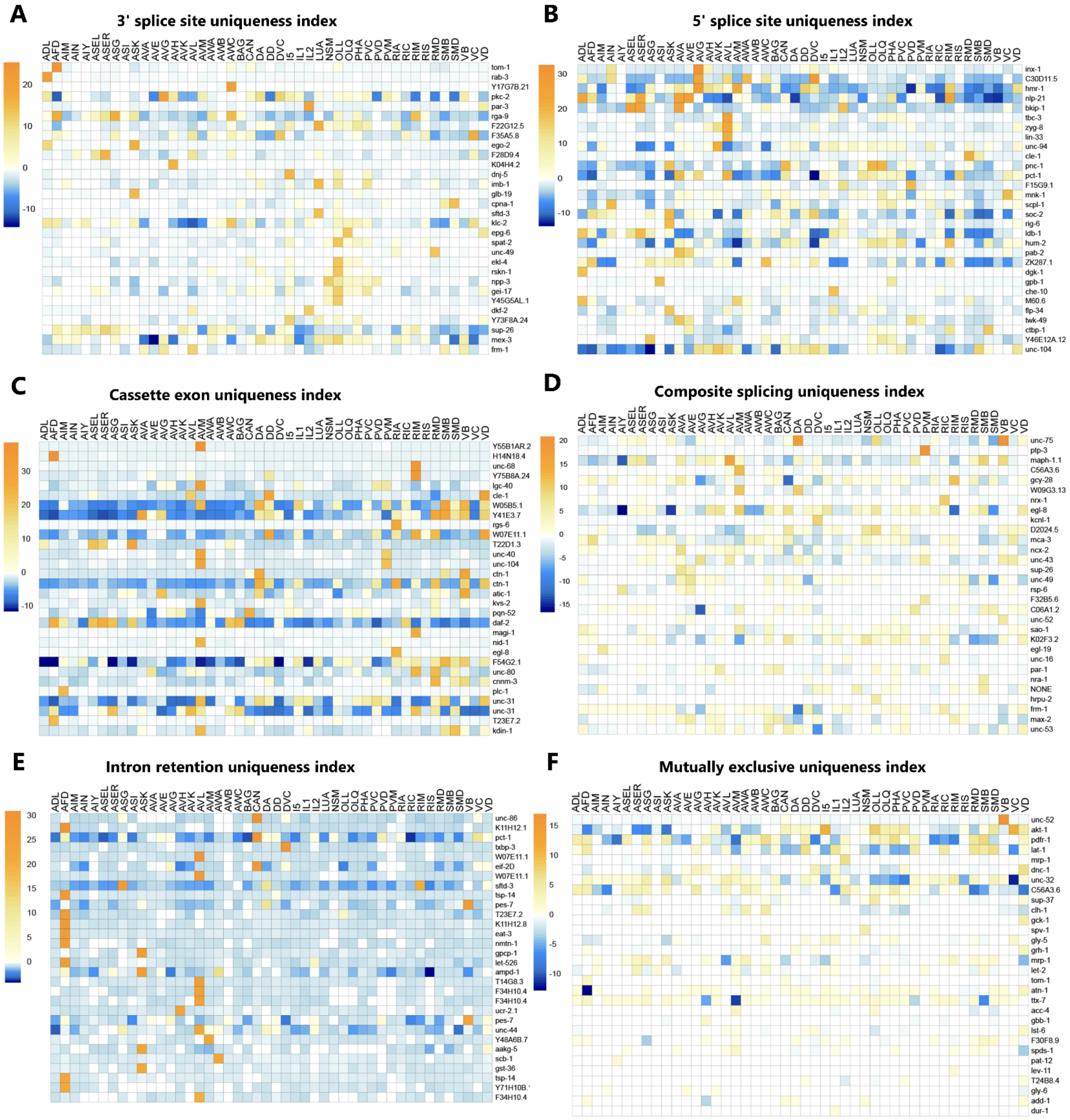

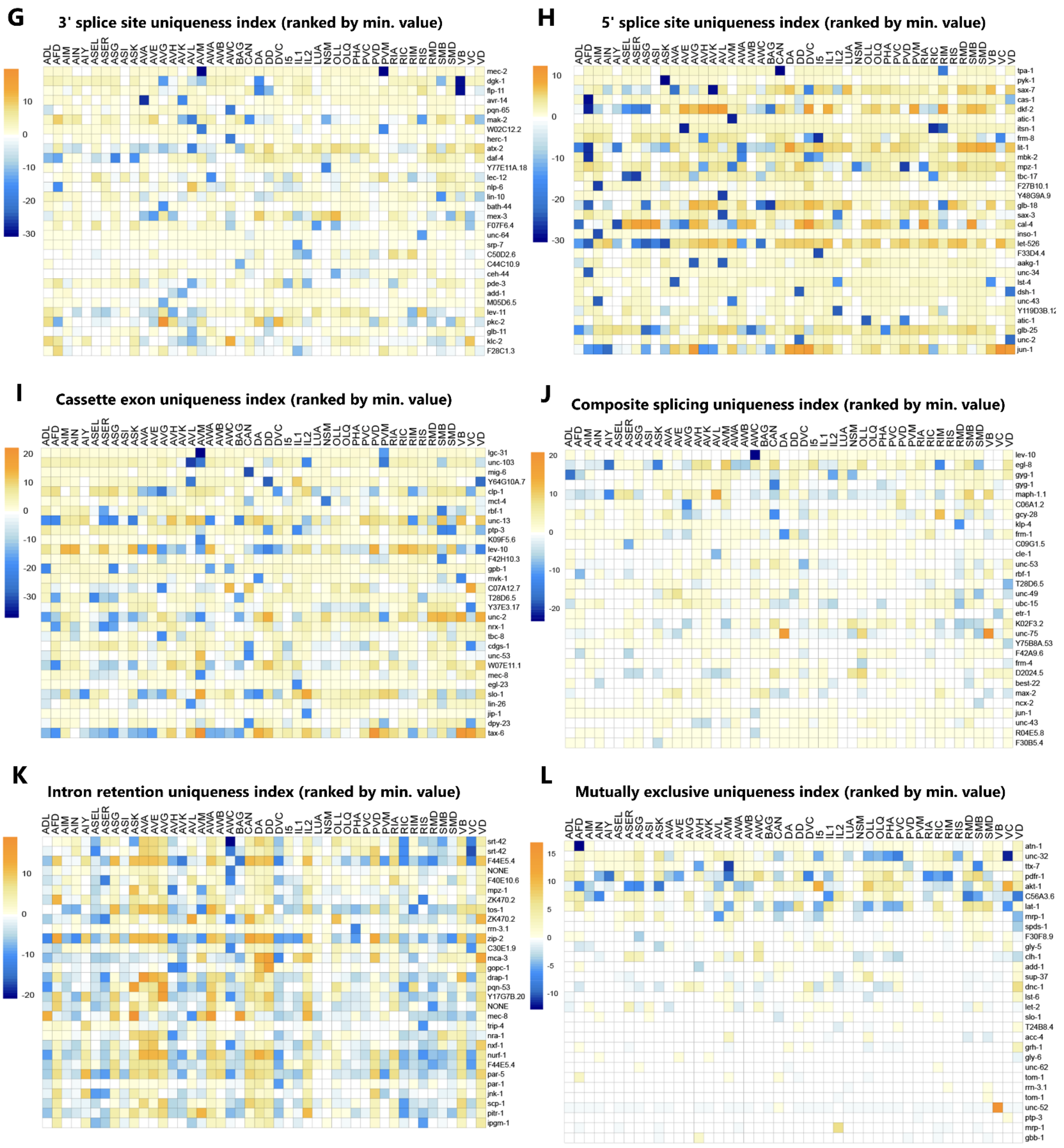

Supplementary Fig. 3: Uniqueness index values for all alternative splicing types. Top 30 uniqueness values, upper 6 heatmaps (A-F) are sorted for highest positive values (corresponding to high PSIs and/or upstream splice site selection), lower 6 heatmaps (G-L) are sorted for most negative values (corresponding to low PSIs and/or downstream splice site selection).

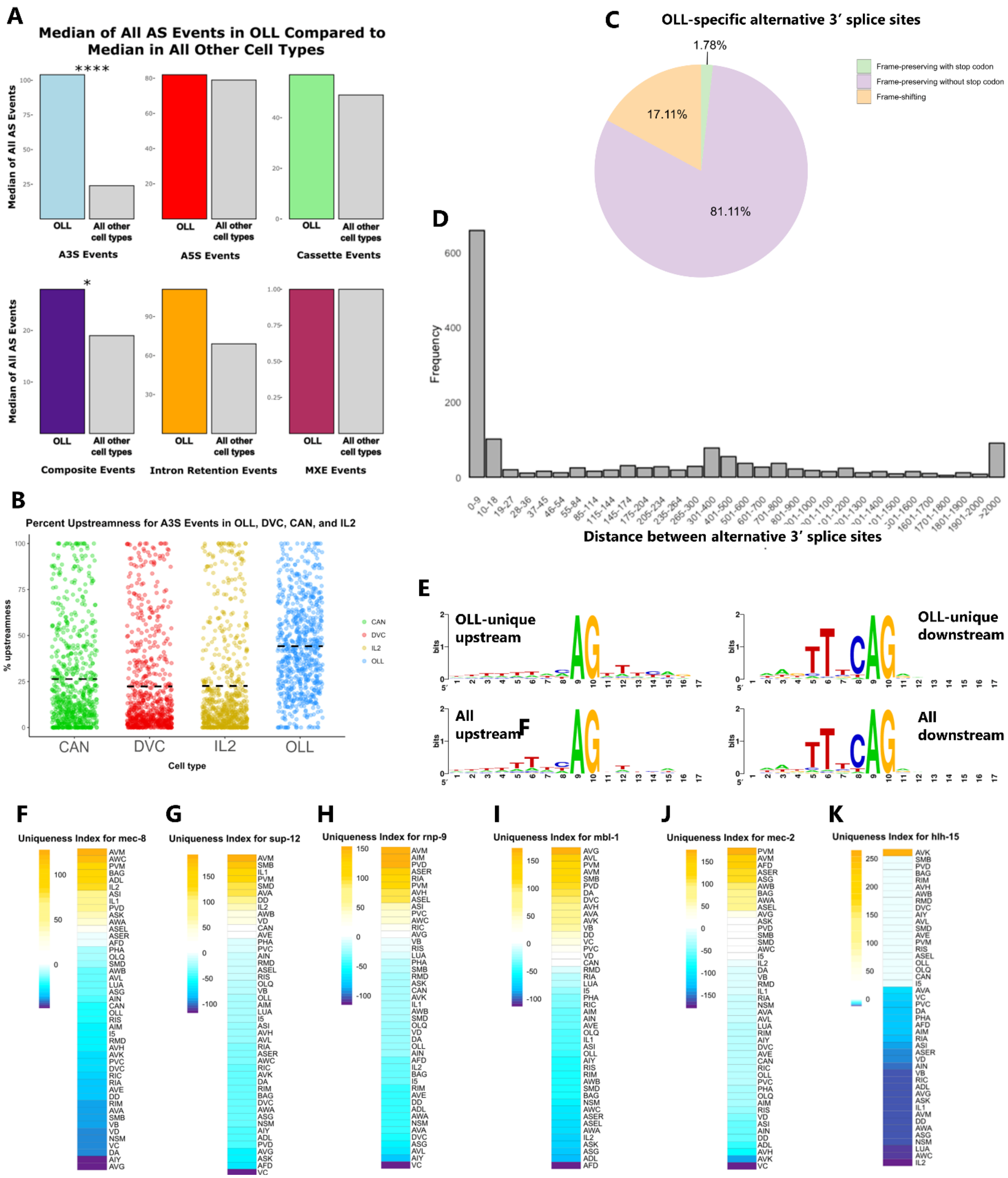

Supplementary Fig. 4: OLL is uniquely enriched for differential 3′ splice site selection, and gene centric uniqueness values. (A) median values of number of differential alternative splicing events between OLL and all other neurons (left, colored bars) versus all other neuron pairwise comparisons (right, gray bars). OLL has much more alternative 3′ splice site selection compared to other neurons, but this is not the case for other types of alternative splicing. **** = t-test, p = 1.697e-07, * = t-test, p = 0.03132. (B) % upstream values for the same 3′ splice sites shown in Figure 4C – each individual dot is a single 3′ splice site choice, and dotted line is population mean. (C) Fraction of OLL-specific alternative 3′ splice site choices that preserve frame and coding sequence (purple) versus those that do not (orange and green). (D) Distribution of distance between alternative

splice sites, revealing a preponderance of small distances (especially 9 nts or fewer). (E) Sequence logos for consensus 3′ splice sites of the upstream and downstream alternative 3′ splice sites. (F-K) Gene centric uniqueness values- handful of selected genes of interest with expression specific to single neurons or subsets of neurons, as revealed by the gene expression uniqueness index. Values for all genes are in Supplemental Spreadsheet S2.

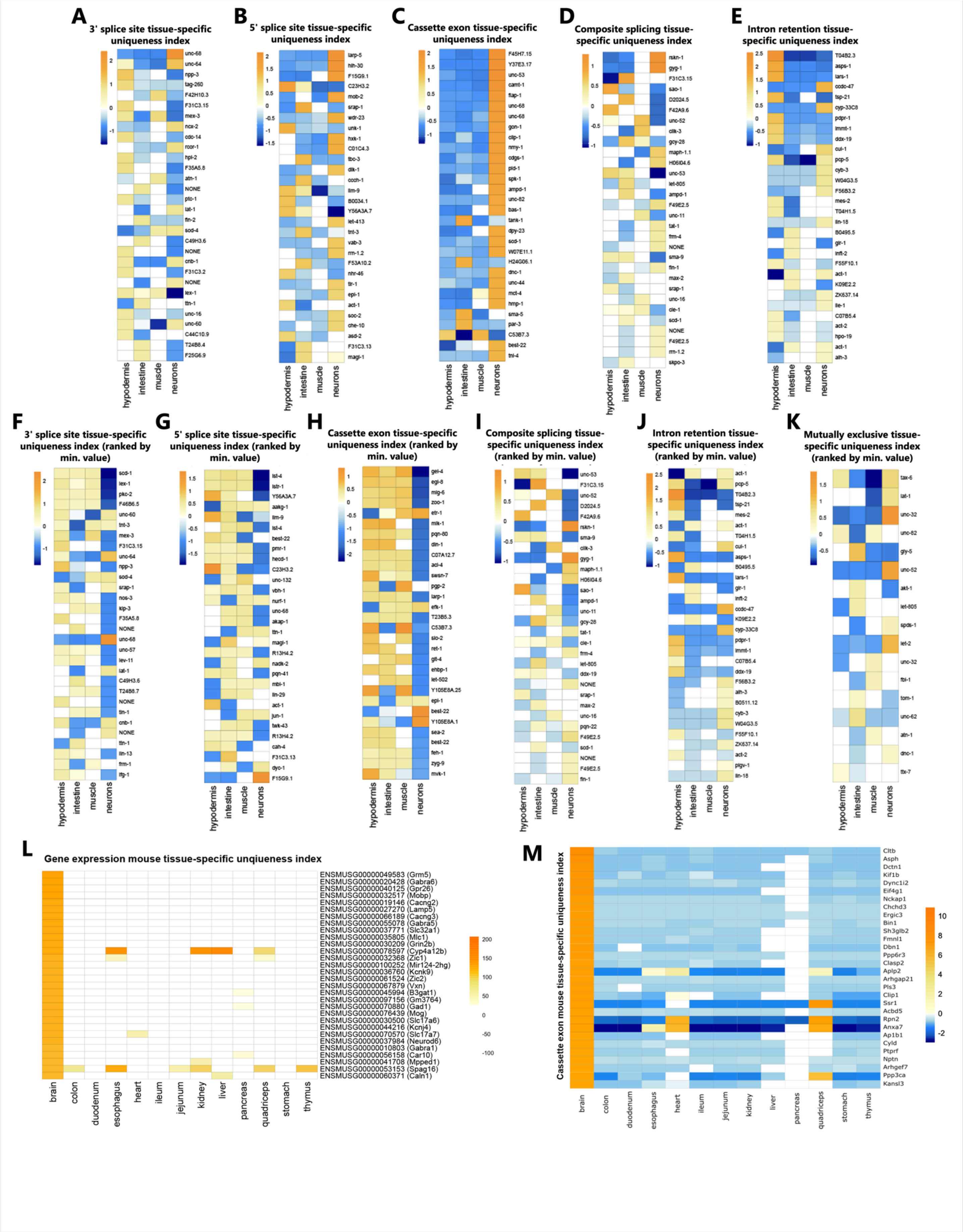
A
3' splice site tissue-specific uniqueness index
B
5' splice site tissue-specific uniqueness index
C
Cassette exon tissue-specific uniqueness index
D
Composite splicing tissue-specific uniqueness index
E
Intron retention tissue-specific uniqueness index
F
3' splice site tissue-specific uniqueness index (ranked by min. value)
G
5' splice site tissue-specific uniqueness index (ranked by min. value)
H
Cassette exon tissue-specific uniqueness index (ranked by min. value)
I
Composite splicing tissue-specific uniqueness index (ranked by min. value)
J
Intron retention tissue-specific uniqueness index (ranked by min. value)
K
Mutually exclusive tissue-specific uniqueness index (ranked by min. value)
hypodermis
intestine
muscle
neurons
L
Gene expression mouse tissue-specific unqiueness index
ENSMUSG00000049583 (Grm5)
ENSMUSG00000020428 (Gabra6)
ENSMUSG00000040125 (Gpr26)
ENSMUSG00000032517 (Mobp)
ENSMUSG00000019146 (Cacng2)
ENSMUSG00000027270 (Lamp5)
ENSMUSG00000066189 (Cacng3)
ENSMUSG00000055078 (Gabra5)
ENSMUSG00000037771 (Slc32a1)
ENSMUSG00000035805 (Mlc1)
ENSMUSG00000030209 (Grin2b)
ENSMUSG00000078597 (Cyp4a12b)
ENSMUSG00000032368 (Zic1)
ENSMUSG00000100252 (Mir124-2hg)
ENSMUSG00000036760 (Kcnk9)
ENSMUSG00000061524 (Zic2)
ENSMUSG00000067879 (Vxn)
ENSMUSG00000045994 (B3gat1)
ENSMUSG00000097156 (Gm3764)
ENSMUSG00000070880 (Gad1)
ENSMUSG00000076439 (Mog)
ENSMUSG00000030500 (Slc17a6)
ENSMUSG00000044216 (Kcnj4)
ENSMUSG00000070570 (Slc17a7)
ENSMUSG00000037984 (Neurod6)
ENSMUSG00000010803 (Gabra1)
ENSMUSG00000056158 (Car10)
ENSMUSG00000041708 (Mpped1)
ENSMUSG00000053153 (Spag16)
ENSMUSG00000060371 (Caln1)
200
150
100
50
0
-50
-100
brain
colon
duodenum
esophagus
heart
ileum
jejunum
kidney
liver
pancreas
quadriceps
stomach
thymus
M
Cassette exon mouse tissue-specific uniqueness index
Cltb
Asph
Dctn1
Kif1b
Dync1i2
Eif4g1
Nckap1
Chchd3
Ergic3
Bin1
Sh3glb2
Fmnl1
Dbn1
Ppp6r3
Clasp2
Aplp2
Arhgap21
Pls3
Clip1
Ssr1
Acbd5
Rpn2
Anxa7
Ap1b1
Cyld
Ptprf
Nptn
Arhgef7
Ppp3ca
Kansl3
10
8
6
4
2
0
-2

Supplementary Fig. 5: Uniqueness index values for tissue-specific alternative splicing in worm and mouse. (A-K) Heatmaps as in Figure 5B but expanded to top 30 splicing events. Upper panels (A-E) are sorted for highest positive values (corresponding to high PSIs and/or upstream splice site selection), lower panels (F-K) for most negative values (corresponding to low PSIs and/or downstream splice site selection). Mutually exclusive exons are presented only once because, due to their small number, all of the top values (both positive and negative) appear on a single heatmap. (L-M) Uniqueness index values for tissue-specific mouse data. (L) Brain-specific gene expression profiles. (M) Brain-specific cassette exons uniquely included in brain compared to all other tissues.

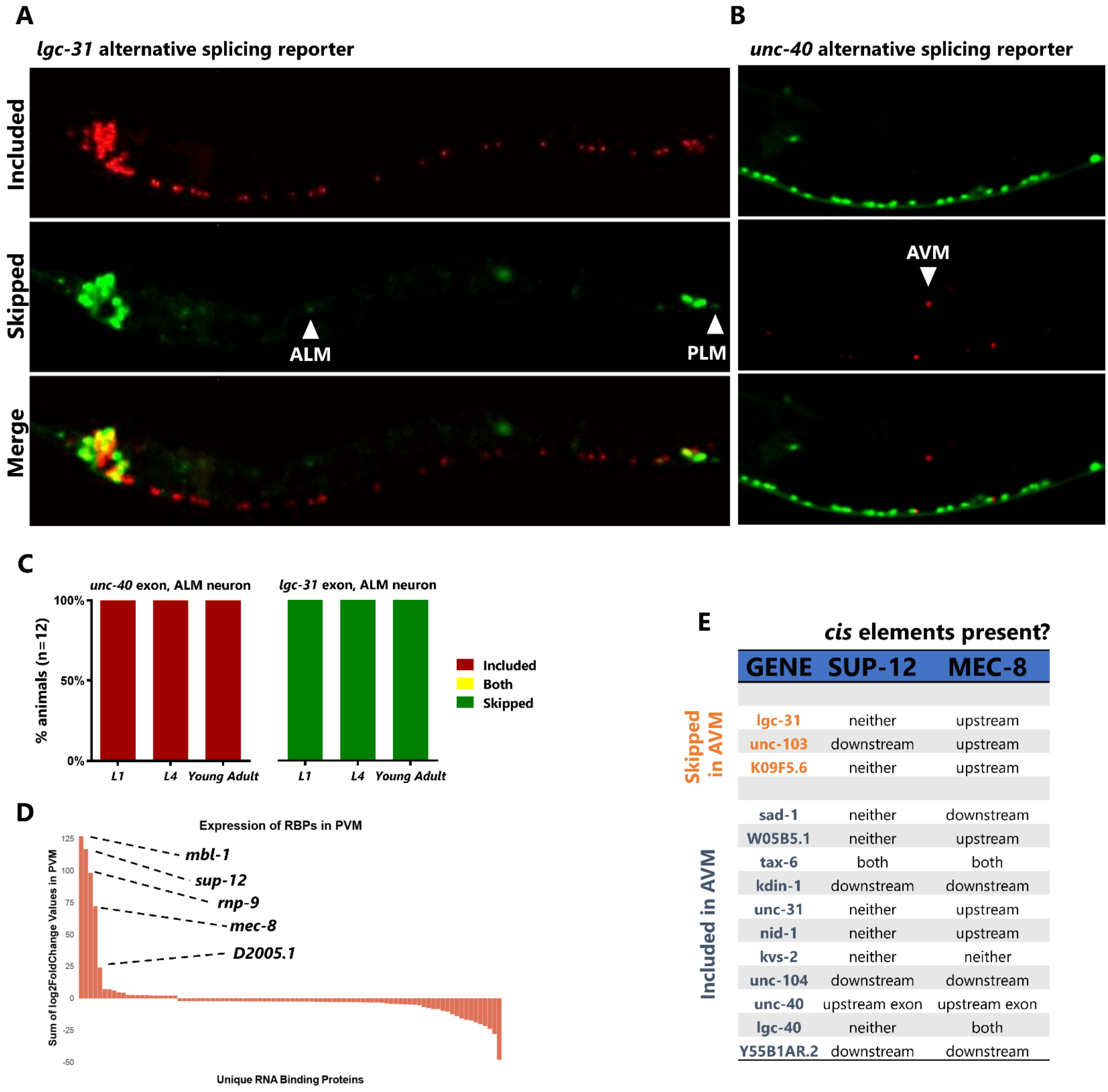

Supplementary Fig. 6: Unique alternative splicing and RNA Binding Protein expression in touch neurons. (A) *lgc-31* splicing reporter, as in 6C for the *unc-40* splicing reporter, shows alternative splicing unique to touch neurons (exon skipping, GFP) compared to many other neurons (exon inclusion, RFP). (B) *unc-40* splicing reporter, as in 6C, but with AVM neuron in focus. Scale bar represents 10 µm. (C) Splicing phenotypes for *unc-40* and *lgc-31* cassette exons are invariant across individuals and across developmental stages (ALM neuron selected due to ease of unambiguous identification). (D) RNA binding protein uniqueness expression levels, as in Figure 6D for AVM, but shown here for PVM. (E) Table showing cassette exons with highest uniqueness values in AVM neuron, and whether predicted *cis* elements for SUP-12 (GUGUG) and MEC-8 (GCAC) are present in the upstream or downstream flanking regions.
