# Supplementary material for: Deep transcriptomics reveals cell-specific isoforms of pan-neuronal genes
Source: Nat Commun. 2025 May 16;16:4507. doi: 10.1038/s41467-025-58296-2 (PMC12084633; doi:10.1038/s41467-025-58296-2)
Supplement: Supplementary file 2 — Description of Additional Supplementary Files [file 41467_2025_58296_MOESM2_ESM.pdf]

File Name: Supplementary Data S1 (counts of differential AS events and genes between cell types).xlsx

Description: This supplementary data file contains the quantities of differentially expressed genes between unique *C. elegans* neuron and tissue types (as determined by DESeq2) as well as the differential counts of alternative splicing events between unique *C. elegans* neuron and tissue types (as determined by JUM).

File Name: Supplementary Data S2 (uniqueness indices).xlsx

Description: This supplementary data file contains the uniqueness indices of each unique *C. elegans* neuron and tissue type for each type of alternative splicing event, as well as a gene-centric (transposed) uniqueness index.

File Name: Supplementary Data S3 (raw percentage usage (averaged) of AS events and normalized counts).xlsx

Description: This supplementary data file contains the raw percentage (%) usage of each unique *C. elegans* neuron and tissue type for each type of alternative splicing event, as well as the normalized (scaled using DESeq2) counts for each individual *C. elegans* neuron and tissue replicate.

File Name: Supplementary Data S4 (raw percentage usage of AS events by individual replicate).xlsx

Description: This supplementary data file contains the raw percentage (%) usage of each individual *C. elegans* neuron and tissue replicate for each type of alternative splicing event.

File Name: Supplementary Data S5 (counts of differential AS events and genes between mouse tissue types).xlsx

Description: This supplementary data file contains the quantities of differentially expressed genes between unique mouse tissue types (as determined by DESeq2) as well as the differential counts of alternative splicing events between unique mouse tissue types (as determined by JUM).

File Name: Supplementary Data S6 (uniqueness indices for mouse tissue types).xlsx

Description: This supplementary data file contains the uniqueness indices of each unique mouse tissue type for each type of alternative splicing event.
