# Supplementary figures and images for: Deep transcriptomics reveals cell-specific isoforms of pan-neuronal genes
Source: Nat Commun. 2025 May 16;16:4507. doi: 10.1038/s41467-025-58296-2 (PMC12084633; doi:10.1038/s41467-025-58296-2)

*Ladder*

Mec-8 rt-  
pcr wild  
type

Mec-8 rt-pcr in mutant  
conditions (unrelated to present  
manuscript)

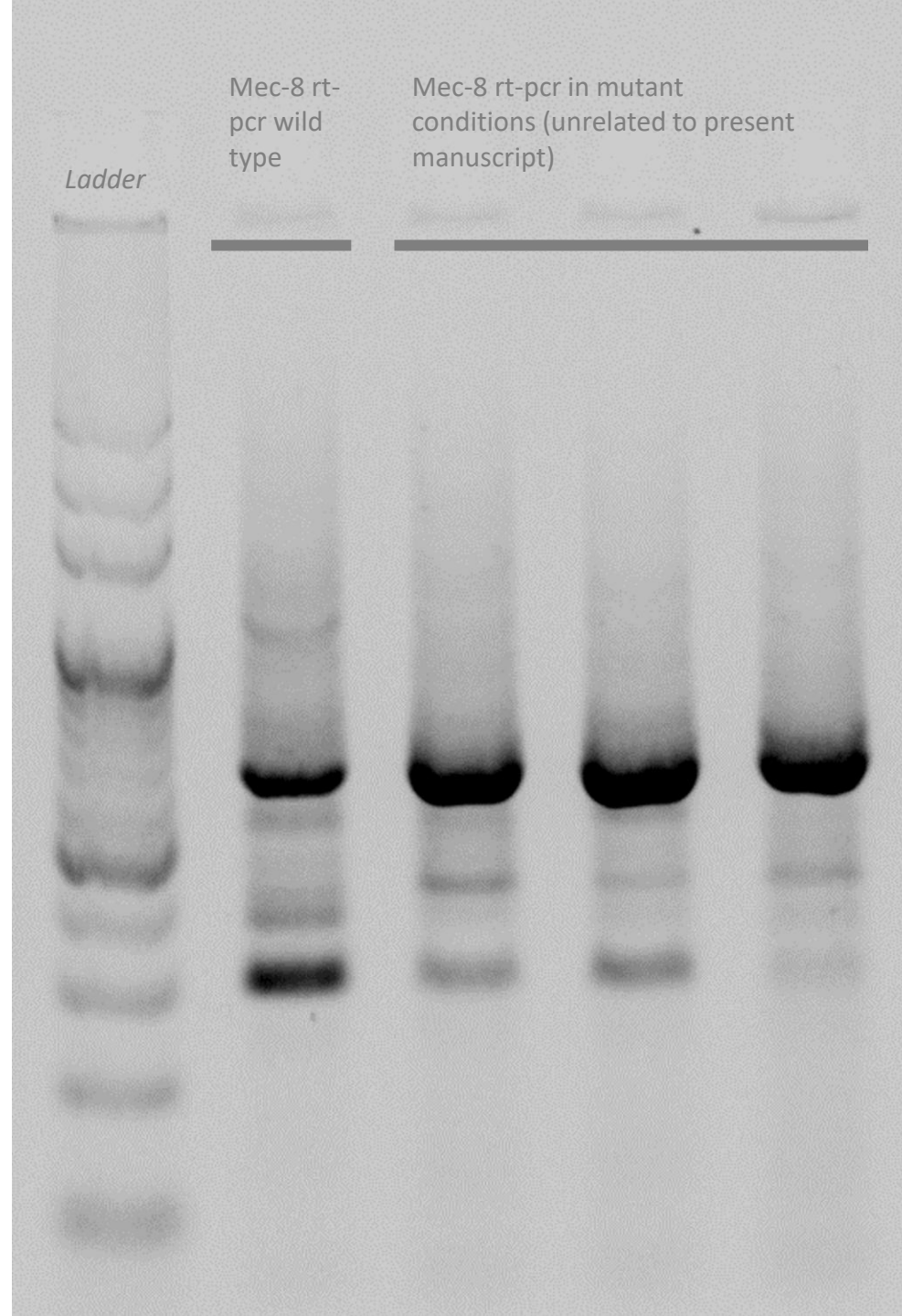

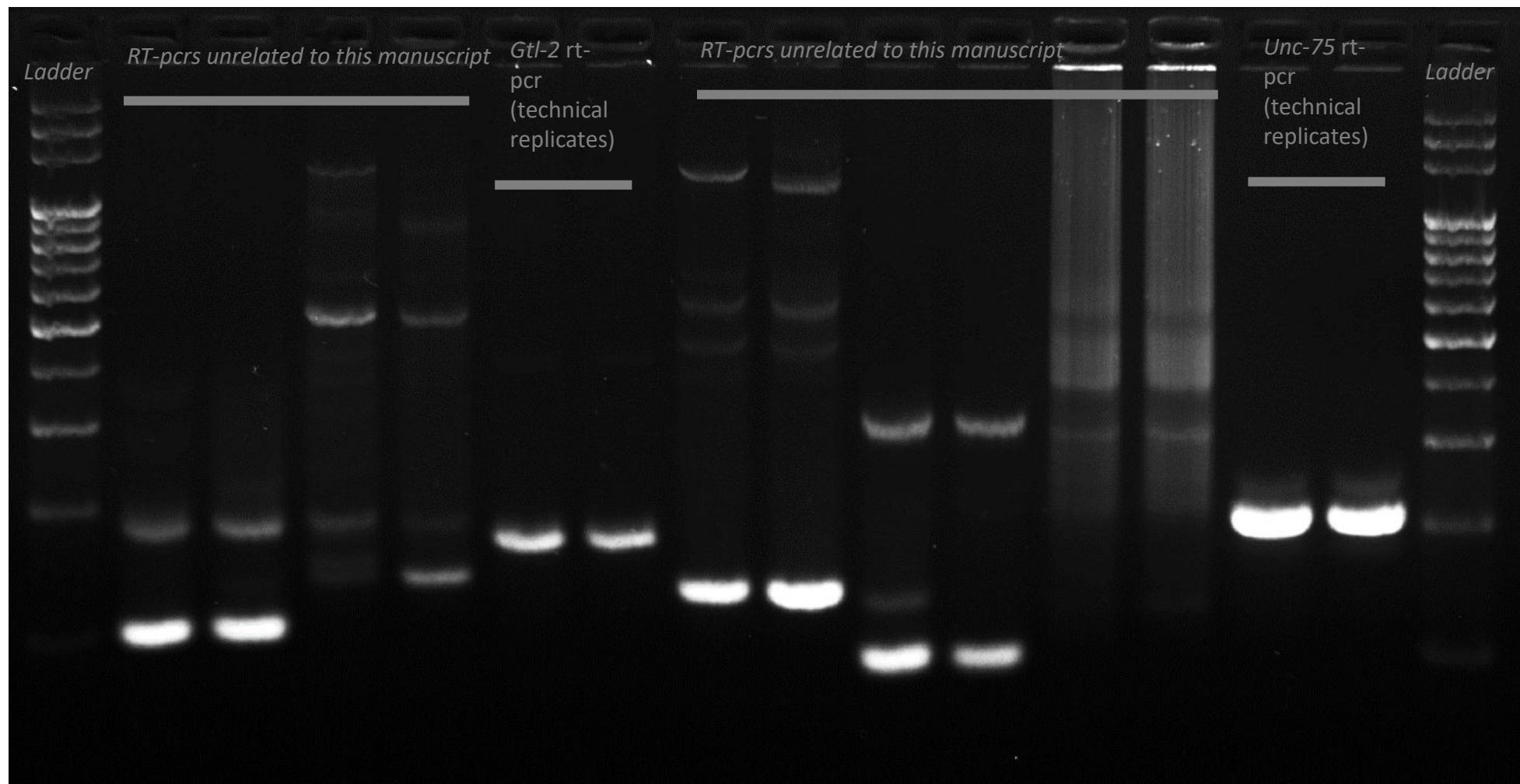

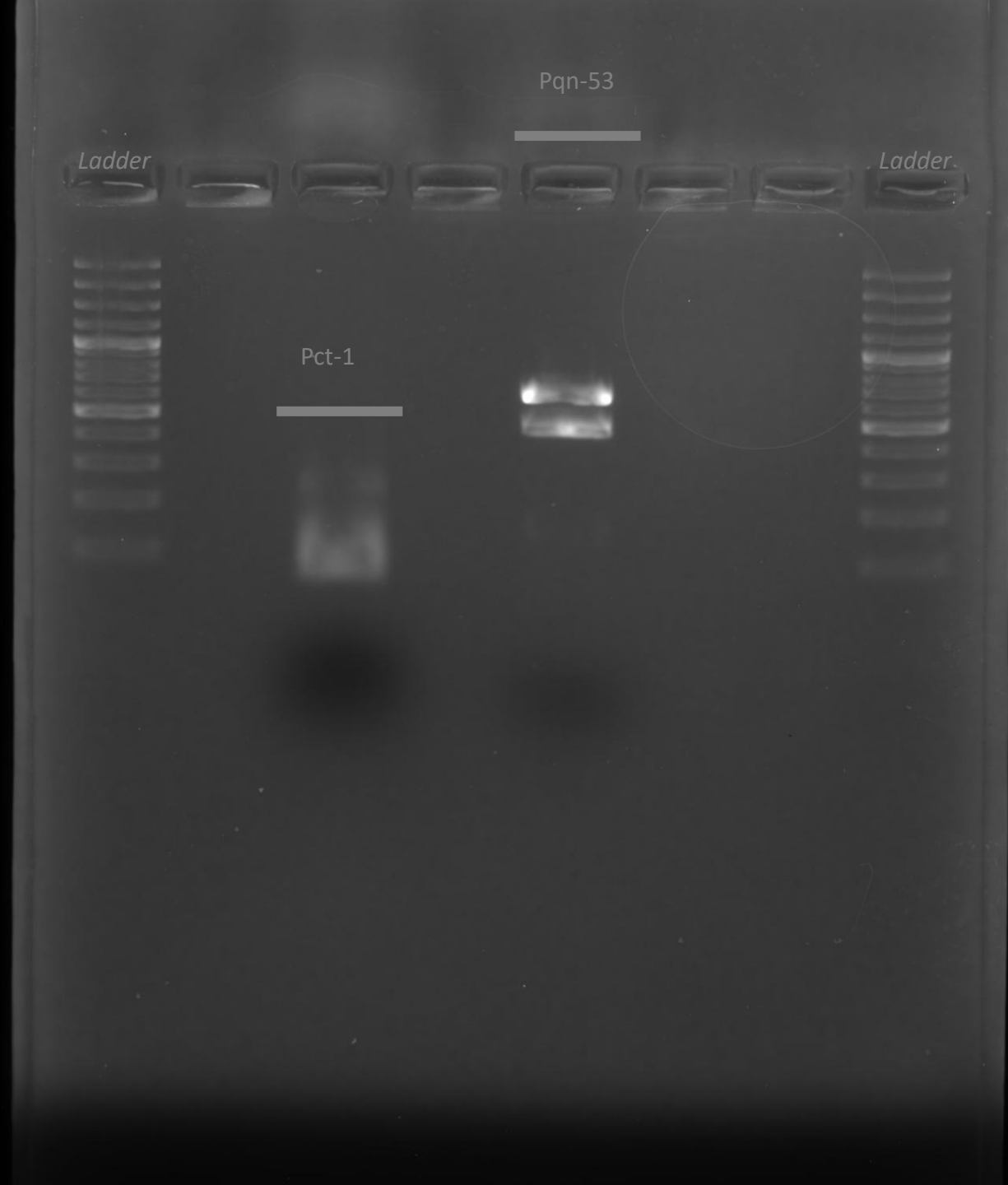

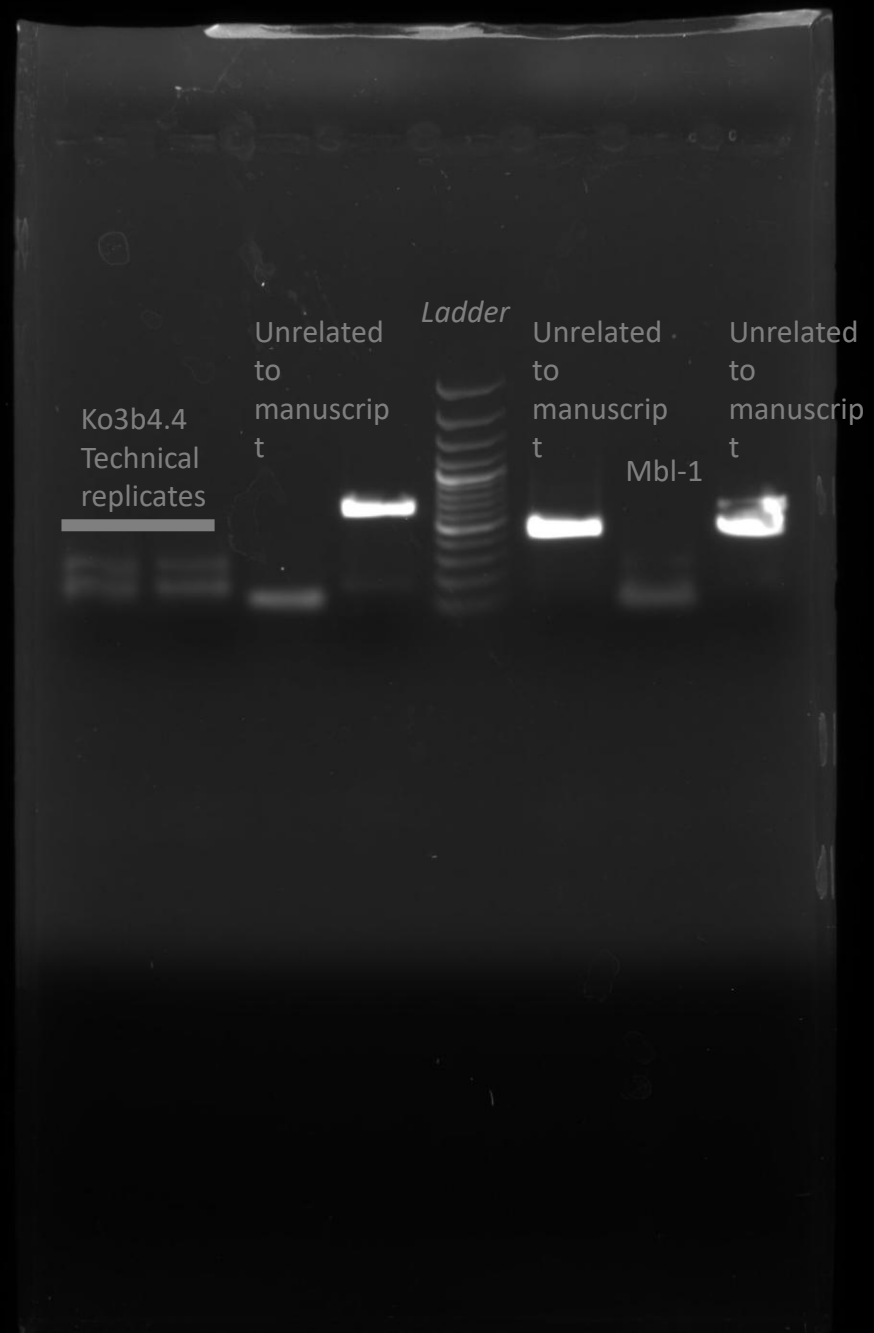

Supplement: Supplementary file 11 — Source Data [file 41467_2025_58296_MOESM11_ESM.pdf]
